# Vanadium complex: an appropriate candidate for killing hepatocellular carcinoma cancerous cells

**DOI:** 10.1007/s10534-018-0139-x

**Published:** 2018-09-25

**Authors:** Hamid Bakhshi Aliabad, Soudeh Khanamani Falahati-pour, Hadis Ahmadirad, Maryam Mohamadi, Mohammad Reza Hajizadeh, Mehdi Mahmoodi

**Affiliations:** 1 Molecular Medicine Research Center, Research Institute of Basic Medical Sciences, Rafsanjan University of Medical Sciences, Rafsanjan, Iran; 2 Pistachio Safety Research Center, Rafsanjan University of Medical Sciences, Rafsanjan, Iran; 3 Department of Clinical Biochemistry, Faculty of Medicine, Rafsanjan University of Medical Sciences, Rafsanjan, Iran; 4 Department of Clinical Biochemistry, Afzalipoor Faculty of Medicine, Kerman University of Medical Sciences, Kerman, Iran

**Keywords:** Vanadium complex, HepG2 cells, L929 cells, Hepatocellular carcinoma, Cytotoxicity

## Abstract

Hepatocellular carcinoma (HCC) is a prevalent human malignancy which its drug resistance is increasing world-wide. This project was designed to assess the anti-cancer effects of 4-bromo-2-(((5-chloro-2-hydroxyphenyl) imino) methyl) phenol ([IV(L)] complex) on the HepG2 cell line and also L929 cells, as normal cells. HepG2 and L929 cells were cultured in RPMI culture medium and the survival rates of the cells were determined after 24 and 48 h using MTT assay to find IC50 concentration of vanadium m, [IV(L)] complex. The early apoptosis and necrosis/late apoptosis were determined by means of annexin V/PI apoptosis detection kit. The results revealed that vanadium m, [IV(L)] complex induce early apoptosis higher in HepG2 cell line than L929 cells. The rates of necrosis/late apoptosis were also induced in HepG2 cells more than L929 cells. Based on the results, vanadium m, [IV(L)] complex might be considered as a safe new drug for treatment of HCC with low side effects on control liver cells.

## Introduction

It has been demonstrated that cancers are the main cause of mortality in the human population (Winters et al. 2017; Tervonen et al. 2017). The Liver is a main organ which can be suffered from cancers which are induced by several factors such as drugs, viruses and chronic inflammation (Ho et al. 2016). Several therapeutic approaches have been introduced for the treatment of cancers including hepatocellular carcinoma (HCC) including radiotherapy, chemotherapy and immunotherapy (Shiba et al. 2017; Obeid et al. 2017). Chemotherapy is a famous approach which is used for the treatment of several cancers including HCC (Le Grazie et al. 2017). However, the current drugs which are used for chemotherapy are associated with several side effects which are derived from their effects on the normal non-cancerous cells (Le Grazie et al. 2017; Ceylan et al. 2015; Clavagnier 2014; Hosseini et al. 2017; Zainodini et al. 2018). Therefore, investigators have been trying to find new therapeutic strategies for cancer treatment with the lowest side effects (Sheikhrezaei et al. 2018; Karimabad et al. 2017; Ramezani et al. 2017; Mohammadizadeh et al. 2018). Based on the fact that HCC is a prevalent cancer word-wide, hence, several studies are designed to introduce new chemotherapy strategies to overcome the disease. Accordingly, several liver cell lines, including HepG2, have been introduced by investigators to examine new drugs for the treatment of HCC (Han et al. 2015). Thus, this cell line has been used in several studies to examine the effectiveness of new anti-cancer drugs.

It has been proposed that vanadium (IV), a metal ion complex, is a suitable candidate for cancer treatment (Nair et al. 2014). It appears that vanadium IV has lower side effects than platinum metal ions (Leon et al. 2017a), hence, recent investigations are focused on the IV complexes to find a suitable drug with the lowest side effects on normal non-cancerous cells. Vanadium compounds as a new class of non-platinum metallodrugs have attracted much attention and large efforts have been made to discover new molecular targets of these compounds (Leon et al. 2017a; León et al. 2016; Sciortino et al. 2018; Ebrahimipour et al. 2015; Abbasi et al. 2017; Hong et al. 2017; Schmidt et al. 2017; Sheikhshoaie et al. 2016; Ebrahimipour et al. 2016; Nair et al. 2016; Takjoo et al. 2013; Takjoo et al. 2017; Heidari et al. 2017; Zabin and Abdelbaset 2016; Mohamadi et al. 2015).

Several mechanisms have been proposed for IV complexes to overcome cancer cells including up-regulation of free radical reactions which is toxic for cancer and normal cells (Wang et al. 2010) altered expression molecules involved in the phosphoinositide-3-kinase-protein kinase B/Akt (PI3K-PKB/Akt), p21 activated protein kinases (PAK), death-associated protein kinase (DAPK), cyclin-dependent kinase (CDK) 4, 6 and 7, Fas-associated protein with death domain (FADD), protein 2-alpha (AP2), and c-Jun N-terminal kinase (JNK) signaling pathways (Leon et al. 2016) and the several molecules such as B cell lymphoma-extra (Bcl-x), Caspase 3 (CASP3), CASP6, CASP7, CASP10 and CASP11 (Leon et al. 2017b). However, the effectiveness of the anti-cancer metals on HCC and also their side effects on normal cells need to be explored by further investigations.

Based on the fact that HepG2 is a well-known cell line for using in HCC related investigations, hence, the main purpose of this study was to appraise the anti-cancerous effects of an IV complex with 4-bromo-2-(((5-chloro-2-hydroxyphenyl) imino) methyl) phenol (L), which abbreviate to [IV(L)] complex, on the HepG2 cell line. On the other hand, due to the various side effects of chemotherapy on the normal cells of the hosts, another goal of this study was to explore the effects of the [IV(L)] complex on the survival and apoptosis of L929, a normal cell line.

## Materials and methods

### Material and instrumentations

#### Cell lines

Human cancerous (HepG2) and mouse fibroblast L929 cell lines have been purchased from Pasture Institute, Tehran, Iran.

#### Cell culture

The HepG2 and L929 cell lines were cultured in the RPMI 1640 culture medium containing 10% of fetal bovine serum (FBS), 100 IU/mL penicillin and 100 µg/L streptomycin, and incubated in a 5% CO_2_ incubator at 37 °C (Memert Company, Germany) (Bagrezaei et al. 2018). To use the cells for examination regarding survival, apoptosis and treatment with the [IV(L)] complex, the growth cells were separated from culture falcons using trypsin-Ethylene diamine tetra acetic acid (EDTA) and centrifuged at 1100 rpm for 5 min. Then, 1 mL fresh RPMI medium was used to make a suspension from the precipitated cells to determine the survived cells percent via MTT assay (Roche CO. Mannheim, Germany) and determination of inhibiting cell growth by 50% (IC50).

### Preparation of 4-bromo-2-(((5-chloro-2-hydroxyphenyl)imino)methyl)phenol (H2L)

Five milliliter of an ethanolic solution containing 0.03 g (0.2 mmol) 2-amino-4-chlorophenol was added to 5 mL solution of equimolar quantity of 2-hydroxy-5-bromobenzaldehyde in ethanol while stirring vigorously. After refluxing the mixture for 30 min, a red precipitate (H2L) was formed. The product was separated by filtration, washed with cold ethanol, and dried in a desiccator over anhydrous CaCl_2_.

m.p.: 199 °C. Anal. Calcd for C1_3_H_9_BrClNO_2_ (326.57 g mol^−1^): C, 47.81; H, 2.87; N, 4.29. Found: C, 47.79; H, 2.82; N, 4.32%. FT-IR (KBr) cm: ν(OH) 3445, ν(N–H) 3131, ν(C = N) 1627, ν(C = Cring) 1580, ν(C–O) 1320, ν(C–Cl) 680, ν(C–Br) 585.

### Preparation of (2,2′-bipyridine)[4-bromo-2-((5-chloro-2- hydroxyphenylimino)methyl)phenol] oxido-vanadium(IV) [VOL(bipy)]

To 6 mL hot methanolic solution of H2L (0.1 mmol, 0.03 g), an equimolar amount of VOSO_4_·3H_2_O (0.1 mmol, 0.02 g) was added. A brown solution was obtained which was refluxed for 1 h. After adding 0.1 mmol (0.02 g) of 2,2′-bipyridine to the mixture, the reflux was continued for further 3 h. Slow evaporation of the solvent resulted in suitable single crystals [VOL(bipy)]. The product was separated, washed with cold ethanol, and dried in a vacuum desiccator over CaCl_2_.

m.p.: 236 °C. Molar conductivity (1.0 × 10^3^ M, DMSO): 20.8 Ω^−1^ cm^−2^ mol^−1^. Anal. Calcd for C_24_H_19_BrClN_3_O_4_V (579.72 g mol^−1^): C, 49.72; H, 3.30; N, 7.25. Found: C, 49.67; H, 3.28; N, 7.29%. FT-IR (KBr) cm1: ν(C = N) 1596, ν(C = Cring) 1511 s, ν(C–O) 1289 m, ν(V = O) 949 s, ρ(pyring) 905 m, ν(C–Cl) 701 m, ν(C–Br) 647w.

### FTIR spectral data

**Fig. 1:**
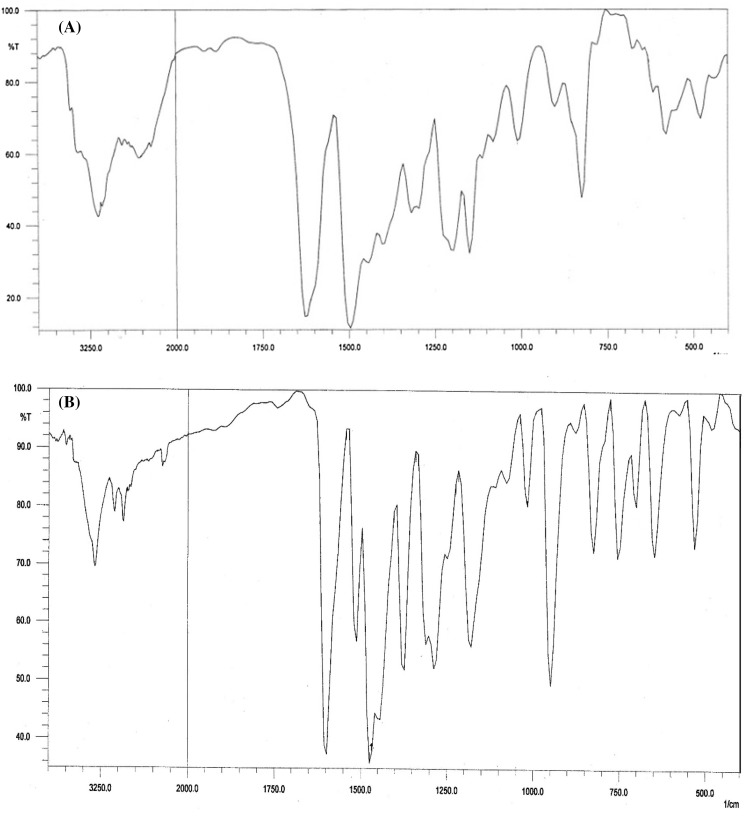
Demonstrates FTIR spectrum of H_2_L (**a**), FTIR spectrum of [IV(L)] (**b**)

In the IR spectrum of H2L, the band appeared at 3131 cm^−1^ was assigned to the NH…O stretching vibration confirming the keto form of the ligand in the solid state. In solution, however, the enol tautomer was the prominent form of the ligand. After the loss of the enolic proton in complexation process, a dianionic ligand was produced (L2-). The band attributed to azomethine moiety was observed at 1627 cm^−1^ in the H2L spectrum and at 1596 cm^−1^ in the [VOL(bipy)] spectrum. This shift suggested the coordination of the azomethine nitrogen to the V(IV) ion. Due to the coordination, the C–O vibration appeared at 1320 cm^−1^ in the ligand shifted to 1289 cm^−1^ in the V(IV) complex confirming the coordination of the oxygen to the metal central ion. The V = O vibration of the vanadyl was observed at 949 cm^−1^ (Fig. 1).

#### 3-(4,5-Dimethylthiazol-2-yl)-2,5-diphenyl tetrazolium bromide (MTT) assay

The MTT cell proliferation assay was used to determine IC50 for the [IV(L)] complex. Briefly, HepG2 and L929 cells were seeded at a density of 1 × 10^4^ cells/well and treated with the [IV(L)] complex at several concentrations for 24 and 48 h. Accordingly, 10 μL MTT (5 mg/mL) (Roche CO. Mannheim, Germany) was added to each wells and incubated at 37 °C for 1 h After solving of formazan crystal in 200 μL of DMSO, the absorbance of the samples was measured at 570 nm with background subtraction at 630 nm. The IC50 of the [IV(L)] complex for HepG2 and L929 cell lines were then calculated to use for analysis of apoptosis by Annexin-V/PI apoptosis detection kit (Ebioscience, CA, USA). To evaluate the time-dependent effect, viability of HepG2 and L929 cells at IC50 value was determined at both 24 and 48 h.

### Apoptosis evaluation

#### Annexin-V-FITC and propidium iodide for analysis of apoptosis

Apoptosis was determined using a commercial annexin V-FITC/PI apoptosis detection kit (eBioscience Co. San Diego, CA, USA). Accordingly, the seeded HepG2 and L929 cells which were pre-treated with IC50 concentration of the [IV(L)] complex for 24 and 48 h, were harvested and washed using cold PBS. The washed cells were incubated with 500 μL binding buffer which contains 1.25 μL of annexin V-FITC and 10 μL of propidium iodide (PI) for 45 min. The stained and un-stained cells were analyzed using Cyflow^®^ space flow cytometer (Partec, Münster, Germany). Flowmax software (Partec) was used for data analysis.

#### Evaluation of morphological changes in the treated cells

The treated cells were also evaluated regarding the alteration in cell morphology, which is a main result of apoptosis. Accordingly, morphological changes on HepG2 and L929 cells after exposure to [IV(L)] complex were examined using an inverted microscope under × 400 magnification. P value of less than 0.05 was considered statistically significant.

#### Statistical analysis

After evaluation of raw data regarding Gaussian distribution, One Way ANOVA under SPSS software version 18 was used to analyze the data.

## Results

The results demonstrated that although, the IC50 for both cell lines at 24 h was the same (100 μg/mL or 0.17 μΜ), it was higher for HepG2 (79 μg/mL or 0.14 μΜ) than L929 (69 μg/mL or 0.12 μΜ) at 48 h. Based on the results it seems that L929 cells, as the normal liver cells, were more sensitive to [IV(L)] complex than the cancerous cells (HepG2). Compared with the clinical drug reference cisplatin with the IC50 of 3.67 μg/mL (0.012 μΜ) at 48 h, these values are large. However, the [IV(L)] complex can be considered and further studied as a potent anticancer agent.

**Fig. 2:**
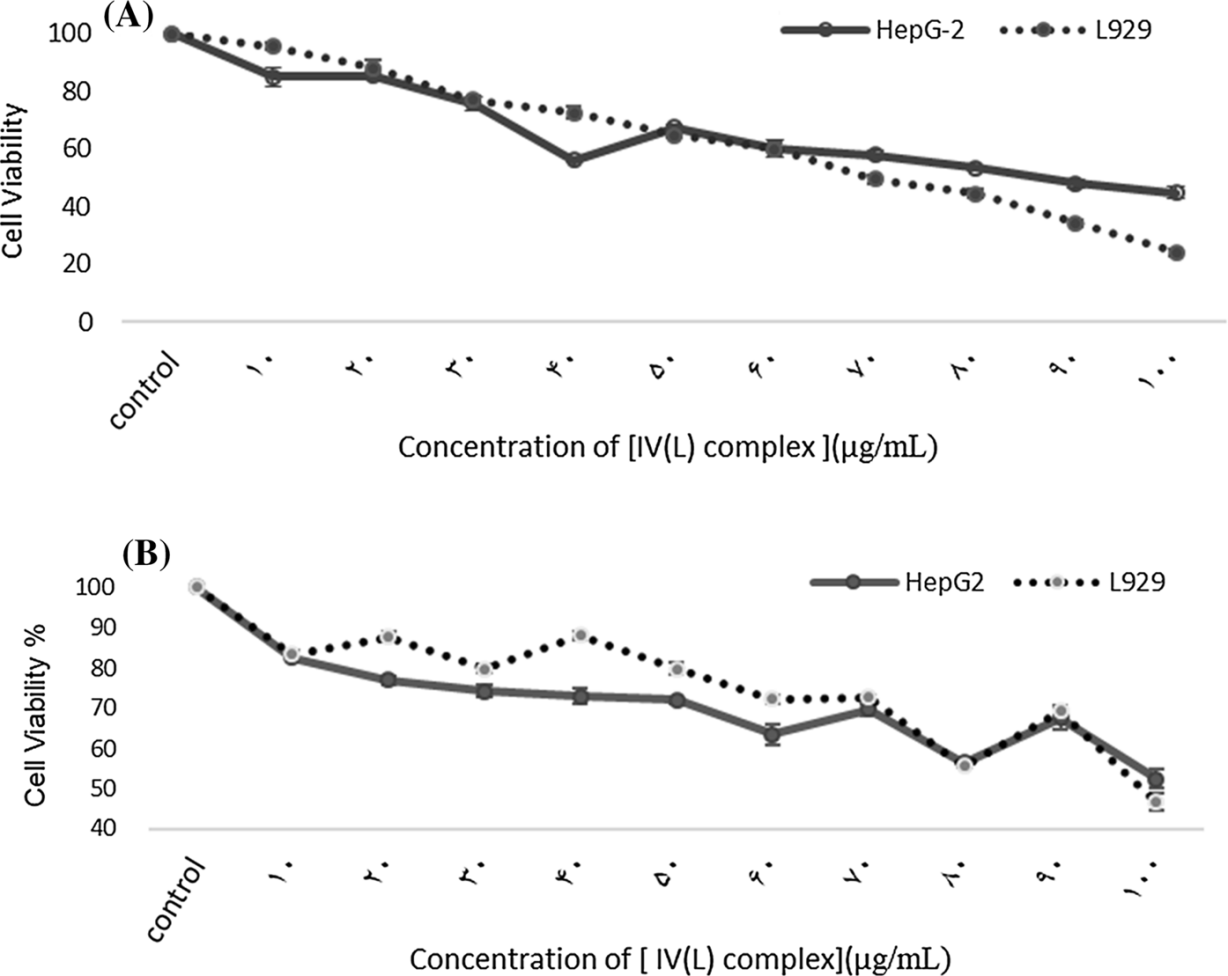
Determination of IC50 in L929 and HepG2 cell lines after 24 and 48 h culture. **a** Illustrates the percent of cell viabilities in L929 and HepG2 cell line at 24 h, **b** illustrates the percent of cell viabilities in L929 and HepG2 cell lines at 48 h, respectively. MTT test revealed that IC50 for L929 cell line at 24 and 48 h were 100 and 69, respectively. IC50 for HepG2 cell line at 24 h was 100 and at 48 h was 79 μg/mL. Cell viability in DMSO and control groups were 100%

**Fig. 3:**
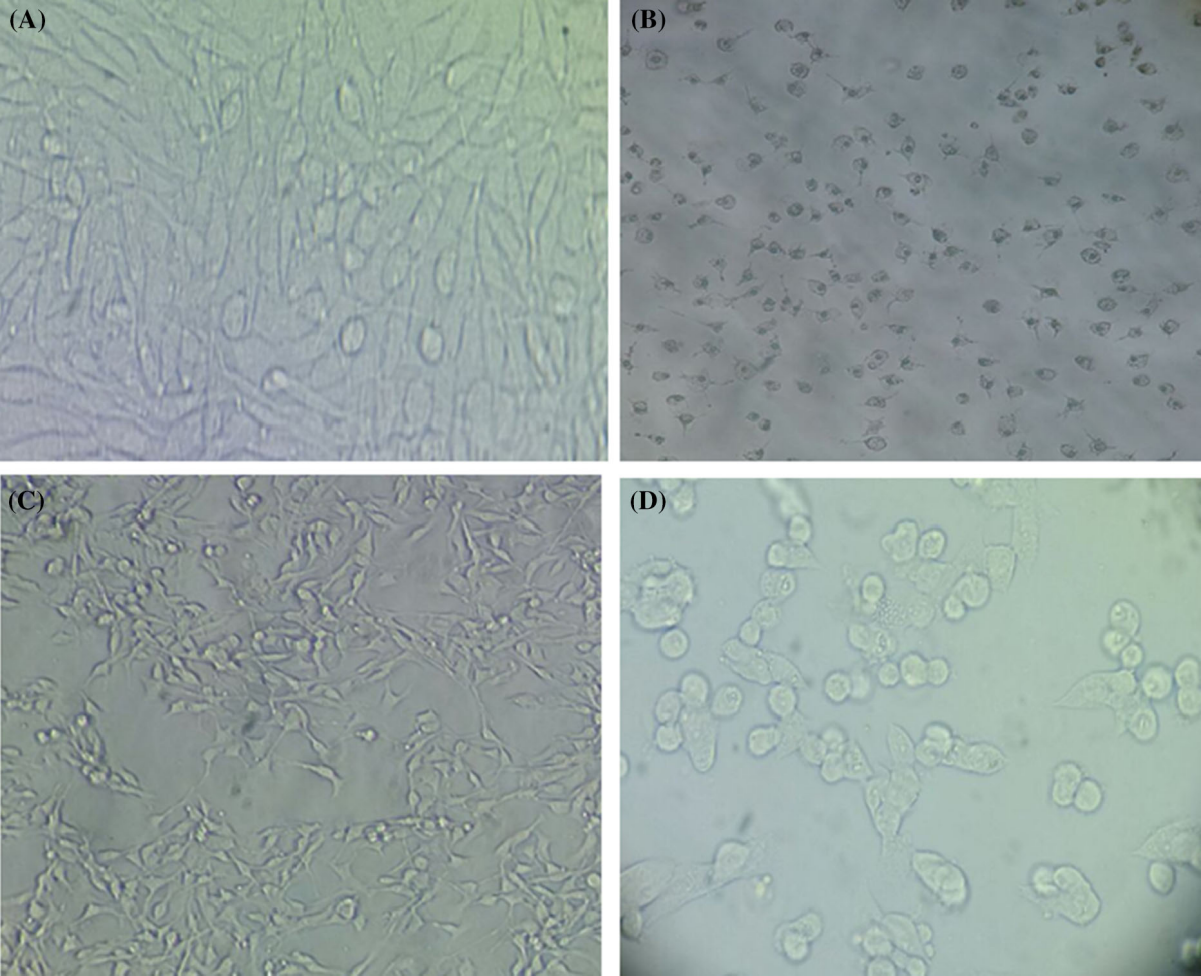
Morphology of L929 and HepG2 cell lines before and after treatment with [IV(L)] complex. [IV(L)] complex induces apoptosis in both cell lines. **a** L929 control before treatment with IV complex. **b** L929 after treatment with [IV(L)] complex. **c** HepG2 control before treatment with [IV(L)] complex and **d** HepG2 after treatment with [IV(L)] complex, respectively

The analysis of results showed that the [IV(L)] complex IC50 values for HepG2 cells at 24 and 48 h were 0.17 μΜ or 100 μg/mL and 0.14 μΜ or 79 μg/mL, respectively. The IC50 value for L929 cells at 24 h was 0.17 μΜ or 100 μg/mL and at 48 h was 0. 12 μΜ or 69 μg/mL (Fig. 2). The morphologic images of L929 and HepG2 cell lines before and after treatment with [IV(L)] complex are presented in the Fig. 3.

Flowcytometry analysis revealed that early and necrosis/late apoptosis rates in the L929 control cells (Untreated) by [IV(L)] complex) were 0.20 and 0.05% after 24 h and 1.09 and 0.01% after 48 h, respectively. However, early and necrosis/late apoptosis rates in the L929 cells which were under treatment of [IV(L)] complex in IC50 concentration were 48.75 and 1.97% after 24 h and 36.85 and 0.10% after 48 h, respectively.

**Fig. 4:**
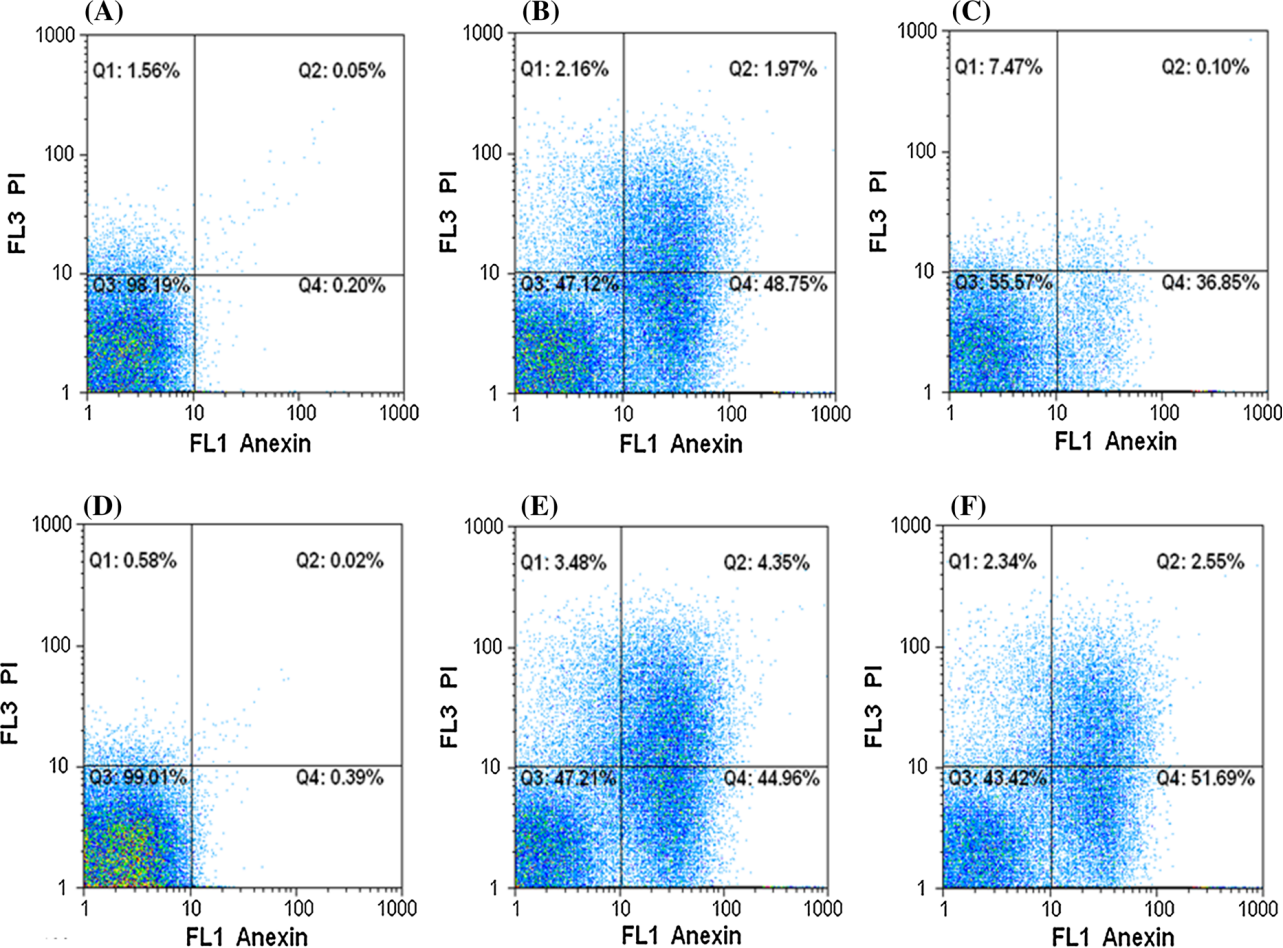
Flow cytometry analysis of HepG2 and L929 cells treated with [IV(L)] complex. Un-treated [IV(L)] complex cells (**a**), HepG2 cells treated at IC50 concentration (100 µg/mL) of [IV(L)] complex for 24 h (**b**), HepG2 treated at IC50 concentration (79 µg/mL) of [IV(L)] complex for 48 h (**c**), un-treated L929 cells (**d**), L929 treated at IC50 concentration (100 µg/mL) of [IV(L)] complex for 24 h (**e**), and L929 cells treated at IC50 concentration (69 µg/mL) of [IV(L)] complex for 48 h (**f**) stained with Annexin V-fluorescein isothiocyanate (FITC) and propidium iodide (PI). Subsequently, apoptotic and necrotic cells were quantified by flow cytometry. The different subpopulations were defined as Q1 annexin V-negative but PI-positive, i.e. necrotic cells; Q2 annexin V/PI double positive, i.e. late apoptotic cells; Q3 annexin V/PI double negative, i.e. normal live cells; Q4 annexin V-positive but PI negative, i.e. apoptotic cells

The early apoptosis rate in HepG2 control cells (without treatment with the [IV(L)] complex) at 24 h was 0.39%, while its necrosis/late rate was 0.02%. Early and necrosis/late apoptosis rates in HepG2 control cells at 48 h were 1.24% and 0.60, respectively. Additionally, early (44.96%) and necrosis/late (4.35%) apoptosis rates in HepG2 treated cells at 24 h were significantly increased by the [IV(L)] complex. Evaluation of early and necrosis/late apoptosis rates in HepG2 treated cells after 48 h also demonstrated that the rates of early (51.69%) and necrosis/late (28.36%) apoptosis rates were increased significantly (Fig. 4 and Table 1).

**Table 1:** Apoptosis percentage at IC50 concentration of [IV(L)] complex (µm/mL)

Apoptosis rates	HepG2		L929	
	24 h (100 µg/mL) (%)	48 h (79 µg/mL) (%)	24 h (100 µg/mL) (%)	48 h (69 µg/mL) (%)
Normal live cells	47.21	43.42	47.12	55.57
Early apoptosis cells	4.35	2.55	1.97	0.10
Late apoptosis cells	44.96	51.69	48.75	36.85
Necrosis cells	3.48	2.34	2.16	7.47

## Discussion

HCC is a prevalent cancer, especially in the viral hepatitis endemic region (Winters et al. 2017; Tervonen et al. 2017). It has been reported that HCC showed drug resistance in some cases (Galun et al. 2017; Dong et al. 2017; Guo et al. 2014) and, hence, investigators are studying new drug candidate to treat the malignancy. However, side effects of the chemical drugs are the main limitations of using these drugs (Yan et al. 2014).

The results demonstrated that although, the IC50 for both cell lines at 24 h was the same (100 μg/mL or 0.17 μΜ), it was higher for HepG2 (79 μg/mL or 0.14 μΜ) than L929 (69 μg/mL or 0.12 μΜ) at 48 h. Based on the results it seems that L929 cells, as the normal liver cells, were more sensitive to [IV(L)] complex than the cancerous cells (HepG2). Compared with the clinical drug reference cisplatin with the IC50 of 3.67 μg/mL (0.012 μΜ) at 48 h, these values are large. However, the [IV(L)] complex can be considered and further studied as a potent anticancer agent (Leon et al. 2017a; León et al. 2016; Sciortino et al. 2018; Ebrahimipour et al. 2015; Abbasi et al. 2017; Hong et al. 2017; Schmidt et al. 2017; Sheikhshoaie et al. 2016; Ebrahimipour et al. 2016; Nair et al. 2016; Takjoo et al. 2013; Takjoo et al. 2017; Heidari et al. 2017; Zabin and Abdelbaset 2016; Mohamadi et al. 2015).

In order to conclude the efficacy of [IV(L)] complex to be considered as a potential candidate for cancer therapy need to have a look to the results obtained from apoptosis and necrosis results. Accordingly, our results showed that the [IV(L)] complex induces early and necrosis/late apoptosis rates in both cancer and normal cell lines. However, the rate of apoptosis, especially early apoptosis, was significantly higher in the HepG2 cells after 48 h incubation with [IV(L)] complex when compared to early apoptosis in the L929, the normal cell line, cells. Due to the fact that induction of early apoptosis is an important key factor for anti-cancer components, hence, based on the results, it appears that [IV(L)] complex can be considered as a potential drug to induce apoptosis in liver tumor cells with lower side effects on normal cells. Additionally, the early apoptosis was higher especially in the HepG2 cell lines when it was incubated with [IV(L)] complex at 48 h. It confirms that more exposure to [IV(L)] complex can induce apoptosis in the cancerous cell lines. Interestingly, the results also showed that necrosis/late apoptosis were also higher in HepG2 cells than L929 cells and in another word, just 0.1% of the L929 cell line suffered from necrosis/late apoptosis (it was 28.36% for HepG2 cells). Based on the fact that necrosis is an inflammatory condition which leads to infiltration of immune cells to the necrotic tissue and consequently leads to immune-related tissue injuries, it may be concluded that [IV(L)] complex probably can be considered as a new HCC treatment strategy because on induction of inflammation in the HCC tissue without injuries to normal cell. To the best of our knowledge, this project is the first investigation on the biological activity of [IV(L)] complex as a novel anti-cancer agent. However, to confirm the anti-cancer effects of the complex, further investigations especially in the in vivo condition need to be performed to prove the anti-cancer roles of [IV(L)] complex and also its safety. In parallel with our results, Nair et al. reported that nicotinoyl hydrazones component of vanadium induces apoptosis in SiHa and HeLa cancerous cell lines via up-regulation of p53, the well-known pro-apoptotic protein (Nair et al. 2014). Wu and colleagues also revealed that Sodium orthovanadate, another component of Vanadium, suppresses human HCC cells in both in vitro and orthotopic in vivo model via modulation of proliferation, cell cycle, apoptosis and autophagy (Wu et al. 2014). The cancer prevention roles played by vanadium were also demonstrated by Mandair et al. (2014) and Bishayee et al. (1997). Interestingly, Kowalski et al. reported that vanadium complexes not only induces apoptosis in human pancreatic ductal adenocarcinoma cell line, but also leads to induction of necroptosis in the cell lines (Kowalski et al. 2017). Our results also showed that the [IV(L)] complex can induce apoptosis in HepG2 cell lines more than L929 cells, and also induces necrosis/late apoptosis in HepG2 cells higher than normal liver cells which can be associated with anti-cancer inflammation. Therefore, our results confirmed the apoptotic and necrotic roles played by vanadium complexes against the cancers. Additionally, based on the results, L929 cells were more sensitive than HepG2 to [IV(L)] complex and have lower IC50 at 48 h incubation. Based on the investigation by Wang et al. (2010) which demonstrated that using antioxidants has synergistic effects with vanadium to kill cancer cells and also reduce their toxicities on human normal cells, it may propose that using [IV(L)] complex in association with antioxidants materials can reduce L929 sensitivities.

Collectively, our results may propose that [IV(L)] complex can be considered as a new strategy for treatment of HCC which need to be evaluated in the in vivo condition. Based on the Wang et al. study, it may also be hypothesized that [IV(L)] complex may be modified by co-administration of antioxidant to be safer than its administration alone.

## Abbreviations

HCC: Hepatocellular carcinoma
Vanadium m, [IV(L)] complex: IV complex with 4-bromo-2-(((5-chloro-2-hydroxyphenyl) imino) methyl) phenol (L)
FBS: Fetal bovine serum
EDTA: Ethylene diamine tetra acetic acid
MTT: 3-(4,5-Dimethylthiazol-2-yl)-2,5- diphenyl tetrazolium bromide
